# Mitochondrial metabolites: undercover signalling molecules

**DOI:** 10.1098/rsfs.2016.0100

**Published:** 2017-04-06

**Authors:** Christian Frezza

**Affiliations:** MRC Cancer Unit, University of Cambridge, Cambridge, UK

**Keywords:** mitochondria, signalling, metabolism

## Abstract

Mitochondria are one of most characterized metabolic hubs of the cell. Here, crucial biochemical reactions occur and most of the cellular adenosine triphosphate (ATP) is produced. In addition, mitochondria act as signalling platforms and communicate with the rest of the cell by modulating calcium fluxes, by producing free radicals, and by releasing bioactive proteins. It is emerging that mitochondrial metabolites can also act as second messengers and can elicit profound (epi)genetic changes. This review describes the many signalling functions of mitochondrial metabolites under normal and stress conditions, focusing on metabolites of the tricarboxylic acid cycle. We provide a new framework for understanding the role of mitochondrial metabolism in cellular pathophysiology.

## 1. Introduction

Mitochondria are intracellular organelles evolved from alpha-proteobacteria engulfed by the ancestor of the eukaryotic cell 2 billion years ago [1]. Although the mechanisms that initiated this symbiosis are still under investigation, it appears that this process was driven by syntrophy, i.e. the beneficial exchange of nutrients between the symbiont, an alpha-proteobacteria, and the host cell, an archeon. In particular, this partnership started under anoxic conditions, where hydrogen molecules released by the mitochondrion's ancestor as by-products of its metabolism were used as an energy substrate by the hydrogen-dependent archeon [2]. The interaction became closer and closer, until when the symbionts were engulfed by the host and became essential partners of the newborn eukaryotic cell. Once engulfed, mitochondria maintained their key functions but they also transferred part of their genome to the nucleus of the host cell [1]. This trade-off was convenient: indeed, mitochondria provided a whole new set of metabolic reactions to the host cell and drove the evolution of the nuclear membrane and other intracellular organelles. At the same time, the host provided protection and a constant food supply to the symbiont [3]. Within this complex relationship, mitochondria evolved several mechanisms of communication with the host, some of which are critical for cell homeostasis. For instance, mitochondria became key players in calcium signalling [4], and in regulation of cell death via the release of pro-apoptotic molecules such as cytochrome *c* [5] when toxic stimuli damage the cell. Furthermore, mitochondria evolved as the main site of production of free radicals, major signalling molecules in the cell [6]. More recently, it has become clear that mitochondrial metabolites, normally considered as mere intermediary for energy generation, can act as signalling molecules by promoting regulatory post-translational modifications on proteins, or via affecting chromatin structure and function [7,8]. This unexpected role of mitochondrial metabolites is the focus of this review.

## 2. The role of mitochondrial metabolites in the communication between mitochondria and the cell

Mitochondria coordinate several metabolic pathways producing metabolites required for cell survival and proliferation. Among these pathways, the tricarboxylic acid (TCA) cycle is a unique metabolic hub. Although the TCA cycle is generally acknowledged for its contribution to energy metabolism and generation of nicotinamide adenine dinucleotide (NADH) to fuel the respiratory chain, this pathway provides the cell with metabolites with unique signalling functions. Here, I describe the most important metabolic nodes within this metabolic hub, focusing on metabolites, rather than on enzymes that produce them, and their involvement in the communication with rest of the cell.

### 2.1. Acetyl CoA

**Figure 1.**
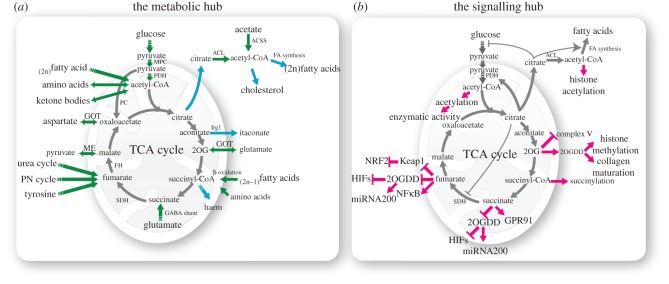
The metabolic and signalling roles of TCA cycle metabolites. (*a*) In this panel, the major sources (green arrows) and fates (blue arrows) of TCA cycle metabolites are indicated. Dotted arrows indicate multi-step reactions, whereas solid lines indicate a single-step reaction. (*b*) Mitochondrial metabolites are also key signalling molecules. Some of the most established signalling functions of TCA cycle metabolites are indicated (purple arrows). ACL, ATP-citrate lyase; ACSS, acetyl-CoA synthetase; FA, fatty acid; FH, fumarate hydratase; GABA, gamma amino butyric acid; GOT, glutamate oxaloacetate transaminase; GPR91, G-protein-coupled receptor 91, also known as succinate receptor; Irg1, immunoresponsive 1 homologue, also known as aconitate dehydrogenase; ME, malic enzyme; MPC, mitochondrial pyruvate carrier; 2OGDH, 2-oxoglutarate dehydrogenase; 2OGDD, 2-oxoglutarate-dependent dioxygenases; PC, pyruvate carboxylase; PDH, pyruvate dehydrogenase complex; PN, purine nucleotides; SDH, succinate dehydrogenase.

The TCA cycle is fuelled by the energy-rich intermediate acetyl-coenzyme A (AcCoA), the activated form of acetate. AcCoA is at a crossroad of multiple metabolic pathways, including carbohydrate, lipids and protein metabolism (figure 1*a*). AcCoA is generated from the oxidation of glucose-derived pyruvate via the pyruvate dehydrogenase complex (PDH), the gatekeeper of carbohydrate metabolism. AcCoA can also be generated from the oxidation of even-numbered fatty acids (β-oxidation) and from the degradation of the amino acids leucine, isoleucine and tryptophan. Finally, recent evidence suggests that under hypoxic conditions [9] and during metabolic stress [10] AcCoA can also be synthesized from acetate and CoA in an ATP-dependent reaction catalysed by the enzyme AcCoA synthetase. From a biosynthetic point of view, AcCoA is the building block for the biosynthesis of lipids, ketone bodies, amino acids, and cholesterol (figure 1*a*). While the biosynthesis of ketone bodies from AcCoA occurs in the mitochondrial matrix, fatty acid synthesis requires the active export of ‘AcCoA’ equivalents in the form of citrate into the cytosol. Here, citrate is converted back to AcCoA and oxaloacetate (OA) by the enzyme ATP-citrate lyase (ACL) and AcCoA can proceed toward fatty acid synthesis. Of note, the cytosolic AcCoA obtained by ACL can also be used for cholesterol biosynthesis, as part of the mevalonate pathway.

Beyond being a metabolic intermediate, AcCoA is an important signalling molecule within the cell (figure 1*b*). Indeed, AcCoA is the substrate for protein lysine acetylation, a post-translational modification that modulates the activity of metabolic enzymes and, in the nucleus, of histones (see [11] for a comprehensive review on acetylation). Interestingly, it has been reported that during cell-cycle progression, PDH complex translocates from the mitochondria to the nucleus, where it can synthesize AcCoA [12]. Although more work is needed to corroborate this important finding, this work suggests that nuclear PDH could contribute to the generation of a local pool of AcCoA for the fine tuning of histone acetylation. For this unanticipated function in cell physiology, AcCoA has been proposed as a ‘sentinel metabolite’: while high nucleo-cytosolic levels of this metabolite are signals for growth and promotes its utilization for lipid synthesis and histone acetylation, under starvation AcCoA is preferentially diverted towards the mitochondria to promote mitochondria-dependent activities such as the synthesis of ATP and ketone bodies [13].

### 2.2. Citrate

The condensation of AcCoA with OA to citrate is catalysed by the enzyme citrate synthase and represents the first committed step of the TCA cycle (figure 1*a*). As indicated above, mitochondrial citrate can be exported in the cytosol, where it can be used as source of AcCoA, making citrate a key lipogenic molecule. Citrate is also an important allosteric regulator of central carbon metabolism [14]. When accumulated, citrate blocks the glycolytic enzyme phosphofructokinase (PFK1), the rate-limiting step of glycolysis. Citrate can also inhibit PFK2, a 6-phosphofructo-2-kinase/fructose-2,6-bisphosphatase bi-functional enzyme that interconverts fructose-6-phosphate in fructose-2,6-bisphosphate, a powerful allosteric activator of PFK1 [15] (figure 1*b*). Furthermore, citrate dampens the activity of the TCA cycle by inhibiting PDH and succinate dehydrogenase (SDH). Ultimately, citrate activates AcCoA carboxylase, the first committed step of fatty acid synthesis, and inhibits the mitochondrial transport of fatty acids catalysed by carnitine palmitoyltransferase 1. Hence, the accumulation of citrate is an important signal for the cell and leads to the inhibition of catabolic reactions (glycolysis, fatty acid oxidation and TCA cycle), favouring anabolism (fatty acid synthesis). It should also be noted that citrate functions as a chelating agent and binds physiologically relevant cations such as Ca^2+^, Zn^2+^ and Mg^2+^. Thanks to this role, citrate acts as a major store of calcium in the bones. Regulating the availability of these ions might have other important consequences for the bioenergetics of the cells. However, this property of citrate has not been extensively investigated.

### 2.3. Cis aconitate/isocitrate

Citrate proceeds in the TCA cycle after conversion to aconitate and to isocitrate, two sequential reactions catalysed by the mitochondrial enzyme aconitase 2. Owing to specific kinetic properties of this enzyme, most cells exhibit a citrate-to-isocitrate ratio of 10 to 1 [16]. Until recently, it was thought that the only role of cis-aconitate in mammalian cells was to serve as a TCA cycle intermediate. However, it was shown that cis-aconitate can also undergo decarboxylation to itaconitate through the activity of the enzyme aconitate decarboxylase, also known as immune-responsive gene 1 protein [17]. Although itaconate is a well-known metabolite in fungi, its role in mammalian pathophysiology has only recently emerged. For instance, upon lipopolysaccharide stimulation, itaconate can accumulate in macrophages, where it exhibits antimicrobial activity [18]. Indeed, itaconitate is a potent inhibitor of isocitrate lyase, a key enzyme of the glyoxylate cycle, a pathway required for the survival of many parasitic pathogens. Interestingly, the ability to degrade itaconate is an important survival strategy for these parasites [19]. Furthermore, recent work supports the idea that itaconate can also inhibit SDH [20], but that it harbours anti-inflammatory effects on macrophages [21].

### 2.4. 2-Oxoglutarate

Isocitrate is converted to 2-oxoglutarate (2OG) by a group of NADH (IDH3) and NAPDH-dependent (IDH2/IDH1) isocitrate dehydrogenase enzymes. 2OG is another convergence point of the TCA cycle. In addition to IDHs, this metabolite can be generated from glutamine-derived glutamate by either glutamate dehydrogenase or glutamate-oxaloacetate aminotransferase (GOT) (figure 1*a*). Given its role as an acceptor of nitrogen in various transamination reactions, 2OG levels probably fluctuate according to the availability of ammonium.

2OG is the co-substrate of 2OG-dependent dioxygenases (2OGDD), enzymes involved in several biological processes, including protein hydroxylation, and DNA and histone demethylation [22]. These enzymes use 2OG and molecular oxygen to oxidize their substrate, producing succinate and carbon dioxide as by-products. Although oxygen has been considered a major player in the regulation of these enzymes, it is now emerging that changes in the ratio between succinate and 2OG can also profoundly affect the enzymatic activity of 2OGDD. Hence, the availability of 2OG represents an interesting link between mitochondrial function and 2OGDD activity. Consistently, it was shown that in naive embryonic stem cells glutamine-derived 2OG maintains an elevated 2OG-to-succinate ratio, promoting histone/DNA demethylation and leading to pluripotency [23]. Furthermore, it was shown that 2OG, by virtue of this epigenetic function, could also promote early differentiation of mouse [24] and human [25] pluripotent stem cells. Finally, via regulation of another type of 2OGDD, the prolyl-4-hydroxylase, 2OG is involved in the generation of 4-hydroxyproline and subsequent collagen maturation, thus affecting extracellular matrix composition [26] (figure 1*b*).

It has been demonstrated recently that 2OG can also control mitochondrial function. Using an elegant approach to identify targets of this metabolite, Chin and co-workers found that 2OG binds to and inhibits ATP Synthase leading to reduced ATP content and decreased oxygen consumption in both *Caenorhabditis elegans* and mammalian cells (figure 1*b*). Via this mechanisms, 2OG was shown to regulate the lifespan of *C. elegans* [27]. Although some criticisms exist on the concentration of 2OG used in this study, which could lead to off-target effects, these results strongly suggest that this metabolite plays a central role in mitochondrial signalling.

### 2.5. Succinyl-CoA

2OG is converted to succinyl-CoA (SuccCoA) via the enzyme 2OG dehydrogenase (2OGDH). SuccCoA is at a crossroad of multiple pathways. Besides being the product of 2OG oxidation, it conveys carbons from the catabolism of odd-numbered fatty acids, and from the degradation of amino acids. Furthermore, SuccCoA reacts with glycine in the first step of haem biosynthesis, a pathway required for the proper function of iron sulfur cluster proteins and for oxygen transport (figure 1*a*).

Besides its role in metabolism, SuccCoA is an important signalling molecule. Indeed, SuccCoA contributes to protein lysine succinylation, a recently discovered post-translational modification [28] (figure 1*b*). Of note, this modification is reversible and several efforts have been made to identify the enzymes involved in its removal. Recently, it was found that both sirtuin 5 [29,30] and sirtuin 7 [31], members of the NAD^+^-dependent class III histone deacetylases sirtuin family, catalyse protein lysine de-succinylation. The consequences of succinylation on protein function are still unclear, but the process is widespread and appears to overlap acetylation. Interestingly, the identification of succinylated histones indicates that this modification could also affect the cell's epigenome [32], expanding the possible mechanisms through which mitochondrial metabolites could perturb chromatin structure and function.

### 2.6. Succinate

SuccCoA is converted to succinate via succinyl-CoA thiokinase. Succinate is also produced from succinic semialdehyde through the enzyme succinate semialdehyde dehydrogenase, an intermediate reaction of the GABA shunt (figure 1*a*). This pathway channels glutamate into the TCA cycle bypassing 2OGDH in conditions when 2OGDH is impaired, such as under oxidative stress. Succinate is converted to fumarate by the TCA cycle enzyme SDH, a key component of the respiratory chain. SDH is emerging as an important regulatory node for mitochondrial function and its mutations have been found to be the leading cause of some types of hereditary and sporadic cancers [7]. Mutations in SDH lead to the aberrant accumulation of succinate, which can accumulate to millimolar levels in the cells [7]. SDH inactivation and ensuing accumulation of succinate can also be caused by lack of oxygen. Indeed, low oxygen levels during ischaemia [33], or during exercise [34], have been shown to increase succinate levels. Of note, the oxidation of succinate in conditions of high proton motive force or highly reduced coenzyme Q, such as those observed in ischaemia, could lead to reverse electron transport (RET), a major source of free radicals in the mitochondria [35]. Succinate-driven RET is responsible for ischaemia reperfusion injury [33] and plays a key role in macrophage polarization [36].

Accumulation of succinate leads to the inhibition of 2OGDD, with profound genetic and epigenetic consequences [7] (figure 1*b*). Seminal work from the Gottlieb laboratory showed that succinate can inhibit prolyl hydroxylases (PHDs), a group of 2OGDD involved in the hydroxylation and subsequent proteasomal degradation of the hypoxia inducible factors [37]. More recent evidence indicates that succinate can also inhibit 2OGDD involved in histone and DNA demethylation [38,39]. Considering the competing role of 2OG and succinate towards 2OGDD, it would be interesting to determine how cells balance these two metabolites to govern the cell's epigenome.

Succinate can also act as a hormone-like molecule. Indeed, succinate accumulated in body fluids under various circumstances, including inflammation or ischaemia, activates the succinate receptor GPR91, with important physiological implications ([40] and reviewed in [41]) (figure 1*b*). For instance, the stimulation of succinate receptors in the kidney leads to the activation of the renin–angiotensin system [42], whereas in the eyes it leads to the production of vascular epithelial growth factor A to support vascularization during development or after injury [43]. Together, these results suggest that succinate has important functions in the cells, well beyond its role as a TCA cycle intermediate.

### 2.7. Fumarate

Fumarate represents another node of the TCA cycle. Besides being generated in the mitochondria by SDH, other metabolic pathways funnel fumarate into the TCA cycle. Indeed, fumarate is a breakdown product of tyrosine metabolism, and it is produced from the urea and purine nucleotide cycles (figure 1*a*). Fumarate is converted to malate by the enzyme fumarate hydratase (FH). Fumarate can accumulate as a consequence of FH inactivating mutations, as in the genetic disorder fumaric aciduria (OMIM 606812), and in the hereditary cancer syndrome hereditary leiomyomatosis and renal cell cancer (OMIM 150800). Here, the loss of FH leads to the accumulation of millimolar levels of fumarate, causing profound alterations of mitochondrial function and cell metabolism [7]. Similar to succinate, the accumulation of fumarate leads to the inhibition of 2OGDD, including PHDs and histone and DNA demethylases [38,39] (figure 1*b*). Via the inhibition of DNA demethylases, fumarate accumulation was linked to the hypermethylation and subsequent epigenetic suppression of a class of antimetastatic micro RNAs, miR-200, leading to an epithelial-to-mesenchymal transition in epithelial cells [44]. Furthermore, high levels of fumarate lead to protein succination, a post-translational modification of protein cysteine residues [45]. Through succination of Keap1, fumarate leads to the aberrant activation of the antioxidant response mediated by Nrf2 [46,47]. Finally, fumarate was also shown to affect the kinase TBK1, leading to the non-canonical activation of NF-κB signalling [48]. Therefore, similarly to succinate, fumarate appears to be an important mitochondrial messenger, capable of eliciting broad genetic and epigenetic reprogramming in the presence of mitochondrial dysfunction.

### 2.8. Malate

Malate is generated from fumarate by the enzyme FH. Malate can also be generated from pyruvate by malic enzymes (ME) (figure 1*a*). Malate has a key role in energy homeostasis both under aerobic and anaerobic conditions. Under normal oxygen tension, the oxidation of malate to OA provides reducing equivalents from the cytosol to the mitochondria through the malate–aspartate shuttle. Under hypoxia, malate undergoes reduction to fumarate, to sustain the generation of succinate [33]. Malate is also an important metabolite in redox homeostasis. Indeed, its conversion to pyruvate by ME leads to the production of reducing equivalents in the form of NADPH. The regulatory functions of malate are currently unknown.

### 2.9. Oxaloacetate

OA is the last metabolite in the TCA cycle, generated from malate by the enzyme malate dehydrogenase. OA is an important nitrogen acceptor in transamination reactions including GOT, which converts OA to aspartate (figure 1*a*). Therefore, OA is an important source of aspartate, a building block for protein and nucleotide biosynthesis [49]. The pool of OA is also replenished via the carboxylation of pyruvate through the enzyme pyruvate carboxylase (PC). Although this enzyme normally converts OA to pyruvate during gluconeogenesis, it was shown that it fuels the TCA cycle in conditions when other major carbon sources, such as glutamine, are depleted [50]. Another reaction that replenishes OA is the carboxylation of the glycolytic intermediate phosphoenolpyruvate (PEP) through the enzyme phosphoenolpyruvate carbokinase (PEPCK). Similar to PC, PEPCK is involved in the production of PEP from OA during gluconeogenesis; however, recent evidence suggests that this enzyme can also work in the PEP-to-OA direction in cancer cells [51,52]. Besides its role in central carbon metabolism, little is known about possible functions of OA as a signalling molecule. Nevertheless, it is worth noting that OA is an unstable metabolite and it undergoes spontaneous decarboxylation to pyruvate in aqueous solution [53]. It is therefore possible that due to its short half-life, OA has not evolved as an efficient signalling molecule.

## 3. From metabolism to signalling, and back

This brief and TCA cycle-centric overview of mitochondrial metabolism puts forward the view that mitochondrial metabolites have a dual role in the cell: on the one side, they are involved in energy metabolism (figure 1*a*); on the other side, they exhibit important signalling functions (figure 1*b*). Interestingly, the enzymes of the metabolic network are regulated by transcriptional changes and post-translational modifications [54] elicited by the same metabolites they produce. In this scenario, mitochondrial metabolites can be seen as an interface between metabolic and signalling networks, coordinating metabolic activity based on nutrient availability [55]. Some questions arise, though. One question is to what extent metabolic and signalling cascades converge, i.e. whether single metabolites are sufficient to fully recapitulate the function of signalling cascades. Some lines of evidence seem to support this hypothesis. The observation that metabolites such as fumarate [44] and 2-hydroxy-glutarate (2HG) [56] are sufficient to drive global epigenetic changes suggests indeed that metabolites play an important role in dictating the cell's phenotype. However, to date no studies have been performed to comprehensively compare the gene signature triggered by fumarate, succinate, or 2HG, to the ones elicited by the specific inhibition of different 2OGDDs. Another question that remains to be addressed is the dynamics of metabolic signalling. While free radicals produced by mitochondria have, in general, a short half-life and their reactivity is restricted to biomolecules within close proximity [57], it is still unclear how persistent the signal triggered by metabolites is and it probably depends on the metabolic perturbation that changes their levels. Indeed, in the presence of profound metabolic alterations, steady state changes in metabolites levels are likely persistent and could sustain long-term phenotypical changes, as observed in FH and SDH-deficient cells, or in IDH-mutant cells. It would be interesting to investigate how metabolic signalling operates under transient conditions, or when metabolic changes fluctuate due to environmental cues. Finally, one question concerns the evolutionary role of metabolic signalling. It is possible that this type of signalling represents the echo of an ancestral mechanism of communication between the mitochondrion and the host cell, which was eventually backed up by more complex signalling cascades that stabilized or limited the signalling thus created. As indicated above, the evolutionary drive of symbiosis seems to lay on synthrophy, i.e. an active exchange of nutrients between the ancestor of mitochondria and its host. It is possible that besides being used as energy substrates, some of the metabolites released by the symbiont harboured regulatory functions and modified the behaviour of the host cell, cementing their long-distance relationship. This added feature of metabolic intermediates would have represented a substantial evolutionary advantage for the endosymbiont. Finally, it is tempting to speculate that mitochondrial metabolites would act not only as signals for the nucleus, but also for adjacent mitochondria, to direct a convergent response inside the cell. Intriguingly, in prokaryotes, the intercellular communication is achieved via a mechanism called *quorum sensing*, whereby signalling molecules released by the individual cells are used to sense population size, leading to synchronized behaviour [58]. Whether TCA cycle metabolites can also play a role in mitochondrion-to-mitochondrion communication is still an open question and more refined technologies have to be developed to achieve the spatio-temporal resolution to investigate the exchange of metabolites between mitochondria.

Overall, the last decade revealed the unexpected role of mitochondria as key signalling platforms in the cell. Understanding how this signalling machinery operates in the cell and its role in pathophysiological conditions will be the challenge of the next decade.
